# Supplementary material for: Age-Associated Salivary MicroRNA Biomarkers for Oculopharyngeal Muscular Dystrophy
Source: Int J Mol Sci. 2020 Aug 22;21(17):6059. doi: 10.3390/ijms21176059 (PMC7503697; doi:10.3390/ijms21176059)
Supplement: Supplementary file 1 [file ijms-21-06059-s001.zip › Supplementary Materials for proof.docx]

Supplementary material

**Table S1.** Dataset demographics. **A**-**B**. Datasets for RNAseq. **A.** Human. Vastus lateralis. **B.** Mouse. Tibialis anterior samples were collected from a single colony. **C.** Human. Dataset of saliva and serum. * Initial diagnosis 1- Ptosis; 2- dysphagia; 3- leg weakness. Age in humans is in years.

| **A.** |  |  |  |  |  |  |  |
| --- | --- | --- | --- | --- | --- | --- | --- |
| **Controls** | | | **OPMD** | | | |  |
| **Samples ID** | **Age** | **Sex** | **Samples ID** | **Age** | **Sex** | **Alanine Stretch** |  |
| C1 | 52 | F | P1 | 50 | F | 13 |  |
| C2 | 54 | F | P2 | 47 | F | 16 |  |
| C2 | 86 | M | P3 | 55 | F | 16 |  |
| C4 | 50 | F | P4 | 74 | M | 14 |  |
| C5 | 88 | F | P5 | 57 | F | 14 |  |
| C6 | 58 | F | P6 | 57 | F | 14 |  |
| C7 | 43 | F | P7 | 55 | F | 14 |  |
| C8 | 42 | M | P8 | 57 | M | 15 |  |
| C9 | 77 | M | P9 | 60 | F | 13 |  |
| C10 | 73 | M | P10 | 68 | F | 13 |  |
| C11 | 70 | F | P11 | 68 | F | 14 |  |
| C12 | 60 | M | P12 | 62 | F | 13 |  |
| C13 | 68 | F | P13 | 68 | M | 16 |  |
| C14 | 67 | F | P14 | 54 | F | 16 |  |
| C15 | 67 | F | P15 | 66 | M | 13 |  |
|  |  |  | P16 | 74 | M | 14 |  |
| N= 15 | AVR age 63.7 | % (F) 66.7 | N=16 | AVR age 60.5 | % (F) 68.7 |  |  |
|  |  |  |  |  |  |  |  |
| **B.** |  |  |  |  |  |  |  |
| **Group** | **N** | **Age (weeks)** | **Gender** |  |  |  |  |
| FVB | 5 | 12 | M |  |  |  |  |
| A17.1 | 5 | 12 | M |  |  |  |  |
|  |  |  |  |  |  |  |  |
| **C.** |  |  |  |  |  |  |  |
| **Controls** | | | **OPMD** | | | | |
| **Samples ID** | **Age** | **Sex** | **Samples ID** | **Age** | **Sex** | **Alanine stretch** | *** Initial diagnosis** |
| c1 | 57 | F | P23 | 59 | F | 16 | 1 |
| c4 | 60 | F | p27 | 62 | M | 13 | 1 |
| c7 | 63 | M | p28 | 77 | F | 16 | 1 |
| c8 | 51 | F | p34 | 62 | M | 15 | 1 |
| c18 | 73 | M | p37 | 64 | F | 13 | 1 |
| c19 | 59 | F | p38 | 51 | M | 14 | 1 |
| c22 | 78 | M | p39 | 53 | M | 16 | 1 |
| c28 | 79 | M | p43 | 67 | M | 14 | 1 |
| c29 | 55 | F | P47 | 62 | M | 16 | 1 |
| c30 | 84 | M | p24 | 74 | M | 11 | 1 |
| c31 | 53 | F | p30 | 79 | F | 16 | 1 |
| c32 | 47 | M | p29 | 59 | M | 13 | 1 |
| c33 | 76 | M | p20 | 63 | M | 16 | 1 |
| c35 | 59 | M | p1 | 68 | M | 15 | 1 |
| c40 | 56 | M | p11 | 54 | F | 16 | 2 |
| c41 | 53 | M | p12 | 57 | M | 16 | 2 |
| c45 | 59 | M | p14 | 49 | M | 16 | 2 |
| c46 | 71 | M | p15 | 61 | M | 16 | 2 |
|  |  |  | p18 | 75 | F | 11 | 2 |
|  |  |  | p22 | 77 | F | 12 | 2 |
|  |  |  | p25 | 55 | F | 16 | 2 |
|  |  |  | p36 | 55 | F | 14 | 2 |
|  |  |  | p4 | 58 | M | 16 | 2 |
|  |  |  | P42 | 59 | M | 14 | 2 |
|  |  |  | p45 | 64 | F | 16 | 2 |
|  |  |  | P46 | 64 | F | 14 | 2 |
|  |  |  | P7 | 62 | F | 13 | 2 |
|  |  |  | p31 | 53 | M | 16 | 2 |
|  |  |  | p33 | 73 | F | 13 | 2 |
|  |  |  | p6 | 50 | F | 16 | 2 |
|  |  |  | p17 | 73 | M | 16 | 3 |
|  |  |  | p5 | 75 | F | 16 | 3 |
|  |  |  | p19 | 63 | M | 14 | 3 |
| N= 18 | AVR age 62.9 | % (F) 33.3 | N=33 | AVR age 62.9 | % (F) 45.5 |  |  |

**Table S3.** A table summary of miRNAs that are associated with muscle pathologies. Only miRNAs that were found as OPMD-associated (*p* < 0.05, FDR) are listed, dysregulation direction is indicated, and highlighted in grey when opposite to OPMD. Only the significantly deregulated OPMD-miRNAs are shown, behalf miR-206, whose p-value (FDR) was 0.06 (therefore is marked with ^>^). The indicated pathologies are referenced, references covering multiple miRNAs are listed in the headings. Only studies in muscles were considered.

|  | **OPMD** | **Muscular Dystrophies** | **Aging** | **Atrophy & Muscle Wasting** | **Myositis** |
| --- | --- | --- | --- | --- | --- |
| **miR-133a-5p** | down | up [9-12] | down [32] |  | down [34] |
| **miR-133a-3p** | down | up [9-12] | down [32] |  | down [34] |
| **miR-133b-3p** | down | up [9-12] | down [32] |  | down [34] |
| **miR-206-3p** | up^>^ | up [9-12] |  |  |  |
| **miR-29b-3p** | down | down [25] |  | up [28] |  |
| **miR-29c-3p** | down | down [25] |  | down [29] |  |
| **miR-29a-3p** | down | down [25] |  | down [29] |  |
| **miR-146b-5p** | up | up [9-12] | up [32] |  | up [11, 12] |
| **miR-155-5p** | up |  | up [32] |  | up [11, 12] |
| **let-7a-5p** | up |  | up [33] |  |  |
| **let-7c-3p** | up |  | up [33] |  |  |
| **let-7b-5p** | up |  | up [33] |  |  |
| **let-7d-3p** | up |  | up [33] |  |  |
| **miR-195-3p** | up |  | up [33] |  |  |
| **miR-136-3p** | down |  | down [32] |  |  |
| **miR-15a-5p** | down |  |  | down [30] |  |
| **miR-16-5p** | down |  |  | down [30] |  |
| **miR-27b-3p** | down |  | down [32] | down [31] |  |
| **miR-27a-5p** | up |  | down [32] | down [31] |  |
| **miR-23b-5p** | up |  |  | down [31] |  |
| **miR-24-2-5p** | up |  |  | down [31] |  |

**Table S4.** A table summary of miRNAs that are significantly associated with age in muscles from OPMD or controls. An age association was determined with a Pearson correlation (*p* < 0.05). Table shows that average expression levels (AVR CPM), and fold-change and *p*-value (FDR) for the OPMD differential expressed (DE) miRNAs.

|  |  | ***p*-value (Pearson)** | **Correlation Coefficient (*r*)** | **DE OPMD** | |
| --- | --- | --- | --- | --- | --- |
|  | **AVR CPM** | **in OPMD** | | ***p*-value** | **FC** |
| hsa-miR-485-3p | 0.80 | 1.10E-03 | 0.845 | 6.55E-03 | 2.03 |
| hsa-miR-379-5p | 28.43 | 1.40E-02 | 0.714 | 2.72E-04 | 1.79 |
| hsa-miR-382-5p | 7.03 | 1.90E-02 | 0.688 | 4.47E-06 | 2.09 |
| hsa-miR-146b-5p | 36.22 | 2.00E-02 | 0.684 | 1.29E-05 | 1.96 |
| hsa-miR-409-3p | 19.18 | 2.00E-02 | 0.683 | 1.04E-05 | 2.10 |
| hsa-miR-200c-3p | 0.97 | 2.30E-02 | 0.675 | 1.09E-04 | 2.46 |
| hsa-miR-493-5p | 11.48 | 2.60E-02 | 0.663 | 8.49E-07 | 2.56 |
| hsa-miR-432-5p | 1.34 | 2.80E-02 | 0.656 | 2.99E-07 | 2.34 |
| hsa-miR-134-5p | 9.66 | 5.00E-02 | 0.603 | 6.42E-04 | 1.97 |
| hsa-miR-517-3p | 0.15 | 4.90E-02 | -0.604 | 4.76E-06 | 1.97 |
| hsa-miR-518b-3p | 4.18 | 9.10E-03 | -0.741 | 5.04E-04 | 2.79 |
|  |  | **in control** | |  |  |
| hsa-miR-3180-5p | 0.04 | 1.62E-02 | 0.650 | 9.39E-07 | 3.94 |
| hsa-miR-184-3p | 1.36 | 3.29E-02 | 0.573 | 1.76E-05 | 2.63 |
| hsa-miR-7151-5p | 0.53 | 5.43E-03 | 0.721 | 7.10E-04 | 2.04 |
| hsa-miR-671-3p | 4.07 | 3.68E-02 | 0.582 | 3.22E-05 | 1.95 |
| hsa-miR-3688-3p | 0.43 | 3.31E-02 | 0.592 | 1.60E-05 | 0.36 |
| hsa-miR-95-3p | 5.17 | 9.30E-03 | 0.688 | 7.83E-07 | 0.20 |
| hsa-miR-6505-5p | 4.60 | 7.87E-03 | 0.529 |  |  |
| hsa-miR-3148-3p | 4.60 | 3.62E-02 | 0.429 |  |  |
| hsa-miR-9-5p | 0.28 | 4.14E-02 | 0.419 |  |  |
| hsa-miR-503-5p | 20.38 | 4.57E-02 | 0.412 |  |  |
| hsa-miR-221-3p | 0.66 | 4.58E-02 | -0.411 |  |  |
| hsa-miR-499a-3p | 0.15 | 1.91E-02 | -0.475 |  |  |

**Table S5.** An assessment of age or gender correlation with the OPMD-associated miRNAs in saliva and serum. A correlation was determined with a Pearson correlation, the correlation coefficient (*r*) and statistical significance are denoted. NS denotes not significant. The OPMD up- or down-regulated miRNAs in muscles are depicted in red and blue, respectively. The unchanged miRNAs are in grey. A correlation with age was assessed for OPMD or all samples, separately. A correlation with gender was studied only for the OPMD sample.

| **Saliva** | **Age (OPMD)** | **Age (control)** | **Gender (OPMD)** |
| --- | --- | --- | --- |
| **miR-200c-3p** | *r* = 0.44 * | NS | NS |
| **miR-15a-5p** | NS | NS | NS |
| **miR-29c-3p** | NS | NS | NS |
| **let-7i** | NS | NS | NS |
| **miR-451a** | *r* = 0.48 * | NS | NS |
| **Serum** | **age (OPMD)** | **age (all)** | **Gender** |
| **miR-15a-5p** | NS | NS | NS |
| **miR-29c-3p** | NS | NS | NS |
| **miR-451a** | NS | NS | NS |
| **miR-23a-3p** | NS | NS | NS |
| **miR-342-3p** | NS | NS | NS |
| **miR-191-5p** | NS | NS | NS |
| **miR-484** | NS | NS | NS |


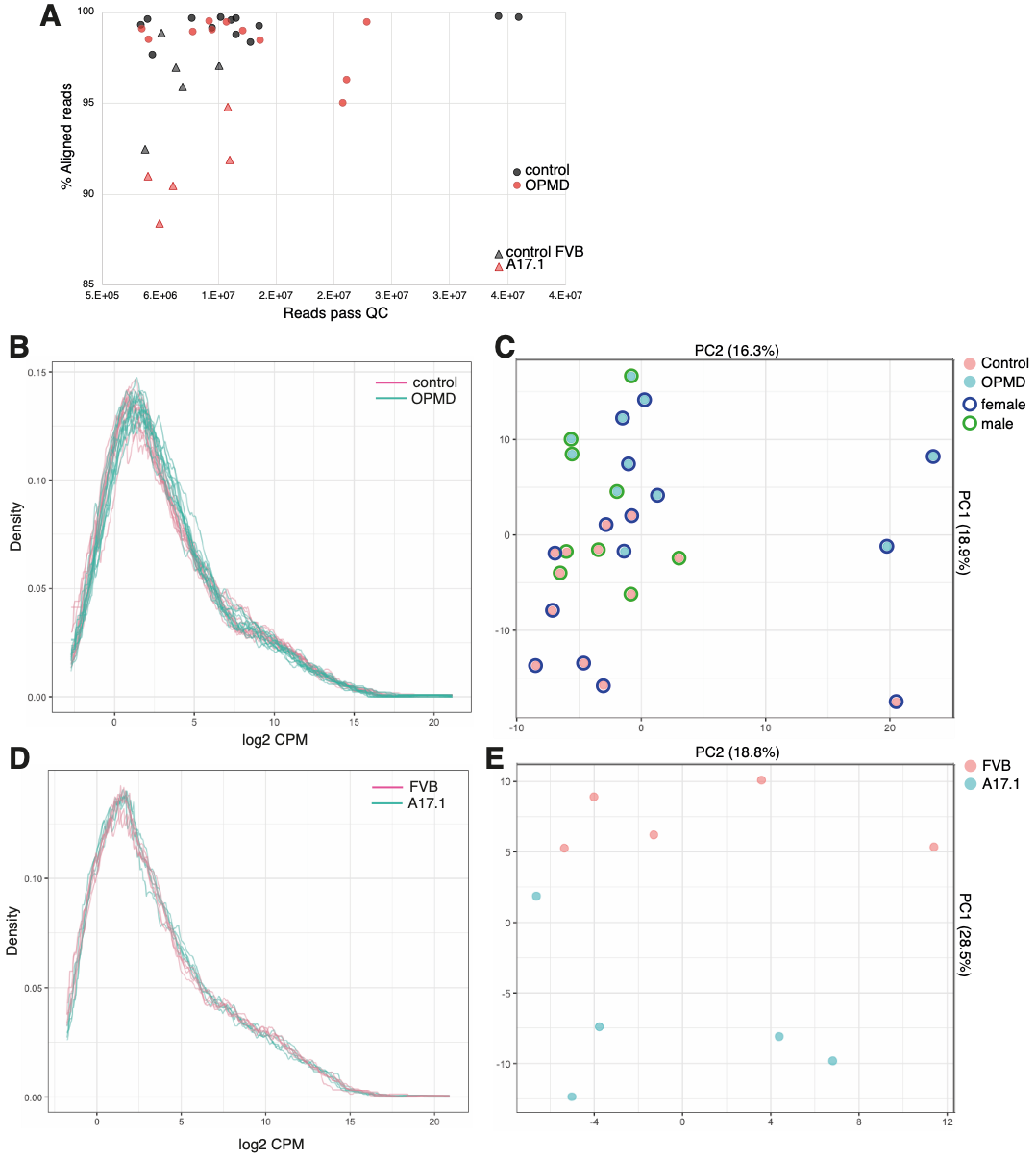


**Figure S1.** RNAseq quality control. (**A**) Scatter plot shows the RNAseq quality control features, reads that pass QC vs. the percentage of aligned reads, in both human and mouse samples. OPMD and the OPMD model, A17.1, are depicted in red. Control samples are depicted in grey. The human samples, OPMD and controls are undistinguishable. QC features of the mouse samples is lower than the human samples. A17.1 had a lower percentage of aligned reads compared with FVB control. (**B**-**C**) human samples, CPM density plot (**B**) and PCA plot (**C**)—C. human samples, CPM density plot (**D**) and PCA plot (**E**).

**Figure S2.** Selection for miRNA candidates in saliva. (**A**) Scatter plot shows the correlation between normalized expression levels of small RNAs in paired muscle and saliva samples from OPMD patients (*n* = 6). RNAs are grouped based on expression levels in saliva: #0 shows RNA with zero reads in saliva, #1-#6 are divided by equal intervals. The correlation is denoted with a line and the 95% confidence in grey. Only for group #6 the correlation was significant, *p*-value and correlation coefficient (*r*) values are denoted. (**B**) Histograms show expression level versus counts in muscles per group. The percentage of RNA from total is depicted under the group number.


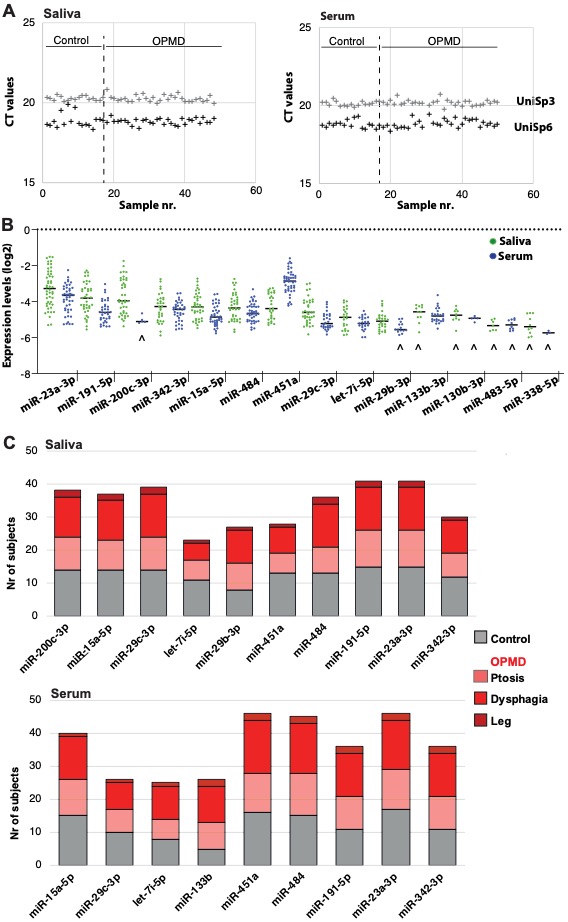


**Figure S3.** qRT-PCR in saliva and serum samples. (**A**) CT levels of spike Unisp3 and Unisp6 in saliva and serum samples. The dashed line separates the controls from OPMD samples. (**B**) Dot bars show means of all expression levels per miRNAs in saliva (green dots) vs. serum (blue dots). Expression levels were normalized to the average of UniSp3 and UniSp6. The ^ sign depicts miRNAs that were excluded due to too few samples with PCR product. (**C**) Chart bars show the numbers of subjects with PCR products (CT < 38) per miRNAs. Within the OPMD group, the number of subjects is noted per an initial symptom. In miR-133b, detection levels in controls are too low and therefore was excluded from analysis.
